# Supplementary material for: Impact of oral diseases on oral health-related quality of life: A systematic review of studies conducted in Latin America and the Caribbean
Source: PLoS One. 2021 Jun 2;16(6):e0252578. doi: 10.1371/journal.pone.0252578 (PMC8171960; doi:10.1371/journal.pone.0252578)
Supplement: S1 Table — (DOCX) [file pone.0252578.s002.docx]

**S1 Table.** Search Strategies.

| **Database** | **PubMed**  **Date:** July, 17 2020 | | **Results** |
| --- | --- | --- | --- |
| **Search Strategy** | #1 | Oral Health[Mesh] | 16,498 |
|  | #2 | Mouth Diseases[Mesh] | 299,395 |
|  | #3 | Oral Health[tiab] | 26,356 |
|  | #4 | Tooth*[tiab] | 99,064 |
|  | #5 | Teeth[tiab] | [114,770](https://pubmed.ncbi.nlm.nih.gov/?term=Teeth%5Btiab%5D&ac=no&sort=relevance) |
|  | #6 | Dental[tiab] | 228,885 |
|  | #7 | Caries[tiab] | 43,909 |
|  | #8 | Denture[tiab] | 16,292 |
|  | #9 | Mouth Disease*[tiab] | 10,738 |
|  | #10 | Periodont*[tiab] | 77,101 |
|  | #11 | Oral Cancer[tiab] | 12,664 |
|  | #12 | Mouth Cancer[tiab] | 364 |
|  | #13 | Oral Tumor*[tiab] | 914 |
|  | #14 | Mouth Tumor*[tiab] | 69 |
|  | #15 | Gingivitis[Mesh] | 11,379 |
|  | #16 | Malocclusion[Mesh] | 33,495 |
|  | #17 | Malocclusion*[tiab] | 12,307 |
|  | #18 | Gingival Disease*[tiab] | 277 |
|  | #19 | Gingivit*[tiab] | 7,962 |
|  | #20 | Crossbite*[tiab] | 2,019 |
|  | #21 | #1 OR #2 OR #3 OR #4 OR #5 OR #6 OR #7 OR #8 OR #9 OR #10 OR #11 OR #12 OR #13 OR #14 OR #15 OR #16 OR #17 OR #18 OR #19 OR #20 | 672,403 |
|  | #22 | Value of Life[Mesh] | 5,706 |
|  | #23 | Quality of Life[Mesh] | 194,599 |
|  | #24 | Quality-Adjusted Life Years[Mesh] | 12,279 |
|  | #25 | DALY*[tiab] | 3,084 |
|  | #26 | QALY*[tiab] | 10,664 |
|  | #27 | Quality-Adjusted[tiab] | 13,572 |
|  | #28 | Related Quality[tiab] | 47,836 |
|  | #29 | HRQOL[tiab] | 17,309 |
|  | #30 | QoL[tiab] | 38,534 |
|  | #31 | "Quality Of Life"[tiab] | 271,785 |
|  | #32 | Dental Impact[tiab] | 50 |
|  | #33 | Social Impact[tiab] | 2,113 |
|  | #34 | Health Impact[tiab] | 8,962 |
|  | #35 | COHQOL[tiab] | 18 |
|  | #36 | CHILD-OIDP[tiab] | 64 |
|  | #37 | OQLQ[tiab] | 72 |
|  | #38 | OHQoL[tiab] | 136 |
|  | #39 | DIDL[tiab] | 17 |
|  | #40 | ECOHIS[tiab] | 149 |
|  | #41 | CPQ*[tiab] | 330 |
|  | #42 | OHIP[tiab] | 1,510 |
|  | #43 | GOHAI[tiab] | 233 |
|  | #44 | #22 OR #23 OR #24 OR #25 OR #26 OR #27 OR #28 OR #29 OR #30 OR #31 OR #32 OR #33 OR #34 OR #35 OR #36 OR #37 OR #38 OR #39 OR #40 OR #41 OR #42 OR #43 | 361,478 |
|  | #45 | Americas[MeSH Termsnoexp] OR America*[tiab] OR Latin America[Mesh] OR Latin America*[tiab] OR Latinamerica*[tiab] OR Latinoamerica*[tiab] OR Latin*[tiab] OR Hispanic Americans[Mesh] OR Hispanic America*[tiab] OR Hispanoamerica*[tiab] OR Iberoamerica*[tiab] OR Ibero Americ*[tiab] OR Panamerican*[tiab] OR Central America[Mesh] OR Central America*[tiab] OR Centroamerica*[tiab] OR Mesoamerica*[tiab] OR Meso America*[tiab] OR Middle America*[tiab] OR South America[Mesh] OR South America*[tiab] OR Southamerica*[tiab] OR Sudamerica*[tiab] OR "America del sur"[tiab] OR Caribbean Region[Mesh] OR Caribbean[tiab] OR Caribe*[tiab] OR West Indies[Mesh] OR West Indi*[tiab] OR Antill*[tiab] OR Indians, South American[Mesh] OR Indians, Central American[Mesh] OR Amerindian*[tiab] OR Indians[tiab] OR American Indian*[tiab] OR Native America*[tiab] OR Patagoni*[tiab] OR Andes[tiab] OR Andean*[tiab] OR Amazon*[tiab] OR Argentin*[ad] OR Argentin*[tiab] OR Argentina[pl] OR Bolivia*[ad] OR Bolivia*[tiab] OR Bolivia[pl] OR Brazil*[ad] OR Brasil*[ad] OR Brazil*[tiab] OR Brasil*[tiab] OR Brazil[pl] OR Colombia*[ad] OR Colombia*[tiab] OR Colombia[pl] OR Chile*[ad] OR Chile*[tiab] OR Chile[pl] OR Ecuador*[ad] OR Ecuator*[ad] OR Ecuador*[tiab] OR Ecuador[pl] OR Guiana*[ad] OR Guiana*[tiab] OR French Guiana[pl] OR Guyan*[ad] OR Guyan*[tiab] OR Guyana[pl] OR Paraguay*[ad] OR Paraguay*[tiab] OR Paraguay[pl] OR Peru*[ad] OR Peru*[tiab] OR Peru[pl] OR Surinam*[ad] OR Surinam*[tiab] OR Suriname[pl] OR Uruguay*[ad] OR Uruguay*[tiab] OR Uruguay[pl] OR Venez*[ad] OR Venez*[tiab] OR Venezuela[pl] OR Belize*[ad] OR Belize*[tiab] OR Belize[pl] OR Costa Ric*[ad] OR Costarric*[ad] OR Costaric*[ad] OR Costa Ric*[tiab] OR Costarric*[tiab] OR Costaric*[tiab] OR Costa Rica[pl] OR Salvador*[ad] OR Salvador*[tiab] OR El Salvador[pl] OR Guatemal*[ad] OR Guatemal*[tiab] OR Guatemala[pl] OR Hondur*[ad] OR Hondur*[tiab] OR Honduras[pl] OR Nicaragu*[ad] OR Nicaragu*[tiab] OR Nicaragua[pl] OR Panam*[ad] OR Panam*[tiab] OR Panama[pl] OR Mexico[Mesh] OR Mexic*[ad] OR Mexic*[tiab] OR Mejic*[tiab] OR Mexico[pl] OR Cuba*[ad] OR Cuba*[tiab] OR Cuba[pl] OR OR Dominic*[ad] OR Dominic*[tiab] OR Dominican Republic[pl] OR Haiti*[ad] OR Haiti*[tiab] OR Haiti[pl] OR Jamaic*[ad] OR Jamaic*[tiab] OR Jamaica[pl] OR Puerto Rico[Mesh] OR Puerto Ric*[tiab] OR Puertorric*[tiab] OR Puertoric*[tiab] | 1,478,008 |
|  | #46 | #21 AND #44 AND #45 | 1,527 |
|  | #47 | #21 AND #44 AND #45 Filters in the last 10 years | 1,235 |

| **Database** | **Embase**  **Date:** July, 17 2020 | | **Results** |
| --- | --- | --- | --- |
| **Search Strategy** | #1 | 'mouth disease'/exp | 604,668 |
|  | #2 | 'oral health'ti,ab | 26,218 |
|  | #3 | tooth*ti,ab | 101,949 |
|  | #4 | dentalti,ab | 231,805 |
|  | #5 | teethti,ab | 110,444 |
|  | #6 | cariesti,ab | 43,483 |
|  | #7 | denture*ti,ab | 24,371 |
|  | #8 | (mouth NEAR/2 disease*)ti,ab | 10,322 |
|  | #9 | periodont*ti,ab | 77,455 |
|  | #10 | 'oral cancer'ti,ab | 14,358 |
|  | #11 | 'mouth cancer'ti,ab | 340 |
|  | #12 | (oral NEAR/2 tumor*)ti,ab | 2,451 |
|  | #13 | (mouth NEAR/2 tumor*)ti,ab | 88 |
|  | #14 | 'gingivitis'/exp | 17,634 |
|  | #15 | gingivit*ti,ab | 8,047 |
|  | #16 | 'malocclusion'/exp | 32,929 |
|  | #17 | malocclusion*ti,ab | 11,600 |
|  | #18 | (gingival NEAR/1 disease*)ti,ab | 354 |
|  | #19 | crossbite*ti,ab | 1,669 |
|  | #20 | #1 OR #2 OR #3 OR #4 OR #5 OR #6 OR #7 OR #8 OR #9 OR #10 OR #11 OR #12 OR #13 OR #14 OR #15 OR #16 OR #17 OR #18 OR #19 | 882,761 |
|  | #21 | 'value of life'ti,ab | 378 |
|  | #22 | 'quality of life'/exp | 488,882 |
|  | #23 | 'quality adjusted life year'/exp | 26,498 |
|  | #24 | qaly*ti,ab | 19,813 |
|  | #25 | 'quality-adjusted'ti,ab | 20,482 |
|  | #26 | 'disability-adjusted life year'/exp | 2,093 |
|  | #27 | dalyti,ab | 2,293 |
|  | #28 | 'related quality'ti,ab | 67,696 |
|  | #29 | hrqolti,ab | 26,536 |
|  | #30 | qolti,ab | 72,599 |
|  | #31 | 'quality of life'ti,ab | 421,057 |
|  | #32 | 'dental impact'ti,ab | 49 |
|  | #33 | 'social impact'ti,ab | 2,924 |
|  | #34 | 'health impact'ti,ab | 10,283 |
|  | #35 | cohqolti,ab | 18 |
|  | #36 | oidpti,ab | 250 |
|  | #37 | oqlqti,ab | 82 |
|  | #38 | ohqolti,ab | 120 |
|  | #39 | didlti,ab | 18 |
|  | #40 | ecohisti,ab | 144 |
|  | #41 | cpq*ti,ab | 626 |
|  | #42 | ohipti,ab | 1,570 |
|  | #43 | gohaiti,ab | 240 |
|  | #44 | #21 OR #22 OR #23 OR #24 OR #25 OR #26 OR #27 OR #28 OR #29 OR #30 OR #31 OR #32 OR #33 OR #34 OR #35 OR #36 OR #37 OR #38 OR #39 OR #40 OR #41 O#42 OR #43 | 606,211 |
|  | #45 | americasti,ab OR 'south and central america'/exp OR ((latin NEAR/1 america*)ti,ab) OR latinamerica*ti,ab OR latinoamerica*ti,ab OR hispanoamericati,ab OR iberoamerica*ti,ab OR ((ibero NEAR/1 americ*)ti,ab) OR panamerica*ti,ab OR ((south NEAR/1 america*)ti,ab) OR southamerica*ti,ab OR sudamerica*ti,ab OR (americati,ab AND delti,ab AND surti,ab) OR ((central NEAR/1 america*)ti,ab) OR centroamerica*ti,ab OR mesoamerica*ti,ab OR ((meso NEAR/1 america*)ti,ab) OR ((middle NEAR/1 america*)ti,ab) OR 'caribbean'/exp OR 'caribbean islands'/exp OR caribbean*ti,ab OR caribe*ti,ab OR ((west NEAR/1 indi*)ti,ab) OR antill*ti,ab OR 'american indian'/exp OR amerindian*ti,ab OR indiansti,ab OR ((native NEAR/1 america*)ti,ab) OR patagoni*ti,ab OR andesti,ab OR andean*ti,ab OR amazon*ti,ab OR 'argentina'/exp OR argentin*ti,ab OR 'bolivia'/exp OR bolivia*ti,ab OR 'brazil'/exp OR brazil*ti,ab OR brasil*ti,ab OR 'colombia'/exp OR colombia*ti,ab OR 'chile'/exp OR chile*ti,ab OR 'ecuador'/exp OR ecuador*ti,ab OR 'french guiana'/exp OR guiana*ti,ab OR 'guyana'/exp OR guyan*ti,ab OR 'paraguay'/exp OR paraguay*ti,ab OR 'peru'/exp OR peru*ti,ab OR 'suriname'/exp OR surinam*ti,ab OR 'uruguay'/exp OR uruguay*ti,ab OR 'venezuela'/exp OR venez*ti,ab OR 'belize'/exp OR beliz*ti,ab OR 'costa rica'/exp OR 'costa rica'ti,ab OR costarric*ti,ab OR costaric*ti,ab OR 'el salvador'/exp OR salvador*ti,ab OR 'guatemala'/exp OR guatemal*ti,ab OR 'honduras'/exp OR hondur*ti,ab OR 'nicaragua'/exp OR nicaragu*ti,ab OR 'panama'/exp OR panam*ti,ab OR 'mexico'/exp OR mexic*ti,ab OR mejic*ti,ab OR 'cuba'/exp OR cuba*ti,ab OR 'dominican republic'/exp OR dominica*ti,ab OR 'haiti'/exp OR haiti*ti,ab OR 'jamaica'/exp OR jamaic*ti,ab OR 'puerto rico'/exp OR ((puerto NEAR/1 ric*)ti,ab) OR puertoric*ti,ab OR puertorric*ti,ab | 513,988 |
|  | #46 | #20 AND #44 AND #45 | 889 |
|  | #47 | #46 AND (2010py OR 2011py OR 2012py OR 2013py OR 2014py OR 2015py OR 2016py OR 2017py OR 2018py OR 2019py OR 2020py) | 758 |

| **Database** | **CINAHL**  **Date:** July, 17 2020 | | **Results** |
| --- | --- | --- | --- |
| **Search Strategy** | S1 | (MH "Oral Health") | 7,902 |
|  | S2 | (MH "Mouth Diseases+") | 40,023 |
|  | S3 | TI (Oral N1 Health) OR AB (Oral N1 Health) | 12,080 |
|  | S4 | TI Tooth* OR AB Tooth* | 19,187 |
|  | S5 | TI Dental OR AB Dental | 47,760 |
|  | S6 | TI Teeth OR AB Teeth | 29,212 |
|  | S7 | TI Caries OR AB Caries | 9,501 |
|  | S8 | TI Denture* OR AB Denture* | 3,707 |
|  | S9 | TI (Mouth N1 Disease*) OR AB (Mouth N1 Disease*) | 427 |
|  | S10 | TI Periodont* OR AB Periodont* | 15,408 |
|  | S11 | TI (Oral N1 Cancer) OR AB (Oral N1 Cancer) | 3,386 |
|  | S12 | TI (Mouth N1 Cancer) OR AB (Mouth N1 Cancer) | 129 |
|  | S13 | TI (Oral N1 Tumor*) OR AB (Oral N1 Tumor*) | 246 |
|  | S14 | TI (Mouth N1 Tumor*) OR AB (Mouth N1 Tumor*) | 6 |
|  | S15 | (MH "Gingivitis+") | 1,393 |
|  | S16 | TI Gingivit* OR AB Gingivit* | 1,501 |
|  | S17 | (MH "Malocclusion+") | 3,003 |
|  | S18 | TI Malocclusion* OR AB Malocclusion* | 1,884 |
|  | S19 | TI (Gingival N1 Disease*) OR AB (Gingival N1 Disease*) | 96 |
|  | S20 | TI Crossbite* OR AB Crossbite* | 304 |
|  | S21 | S1 OR S2 OR S3 OR S4 OR S5 OR S6 OR S7 OR S8 OR S9 OR S10 OR S11 OR S12 OR S13 OR S14 OR S15 OR S16 OR S17 OR S18 OR S19 OR S20 | 104,835 |
|  | S22 | (MH "Economic Value of Life") | 327 |
|  | S23 | (MH "Quality of Life+") | 91,707 |
|  | S24 | (MH "Quality-Adjusted Life Years") | 3,835 |
|  | S25 | TI QALY* OR AB QALY* | 4,353 |
|  | S26 | TI "Quality-Adjusted" OR AB "Quality-Adjusted" | 5,628 |
|  | S27 | TI "Related Quality" OR AB "Related Quality" | 22,333 |
|  | S28 | (MH "Disability-Adjusted Life Years") | 155 |
|  | S29 | TI DALY OR AB DALY | 614 |
|  | S30 | TI "Related Quality" OR AB "Related Quality" | 22,333 |
|  | S31 | TI HRQOL OR AB HRQO | 7,971 |
|  | S32 | TI QoL OR AB QoL | 16,367 |
|  | S33 | TI “Quality Of Life” OR AB “Quality Of Life” | 117,491 |
|  | S34 | TI “Dental Impact” OR AB “Dental Impact” | 24 |
|  | S35 | TI “Social Impact” OR AB “Social Impact” | 758 |
|  | S36 | TI “Health Impact” OR AB “Health Impact” | 3,448 |
|  | S37 | TI COHQOL OR AB COHQOL | 8 |
|  | S38 | TI OIDP OR AB OIDP | 140 |
|  | S39 | TI OQLQ OR AB OQLQ | 25 |
|  | S40 | TI OHQoL OR AB OHQoL | 57 |
|  | S41 | TI DIDL OR AB DIDL | 5 |
|  | S42 | TI ECOHIS OR AB ECOHIS | 89 |
|  | S43 | TI CPQ* OR AB CPQ* | 205 |
|  | S44 | TI OHIP OR AB OHIP | 310 |
|  | S45 | TI GOHAI OR AB GOHAI | 111 |
|  | S46 | S22 OR S23 OR S24 OR S25 OR S26 OR S27 OR S28 OR S29 OR S30 OR S31 OR S32 OR S33 OR S34 OR S35 OR S36 OR S37 OR S38 OR S39 OR S40 OR S41 OR S42 OR S43 OR S44 OR S45 | 150,758 |
|  | S47 | TI (Latin America* OR Latinamerica* OR Latinoamerica* OR Latin* OR Hispanic Americans OR Iberoamerica* OR Ibero Americ* OR Panamerican* OR Central America* OR Centroamerica* OR Mesoamerica* OR Meso America* OR | 39,162 |
|  |  | Middle America* OR South America* OR Southamerica* OR Sudamerica* OR America del sur OR Caribbean OR Caribe* OR West Indi* OR Antill* OR Amerindian* OR Indians OR American Indian* OR Native America* OR Patagoni* OR Andes OR Andean* OR Amazon* OR Argentin* OR Bolivia* OR Brazil* OR Brasil* Colombia* OR Colombia* OR Colombia OR Chile* OR Ecuador* OR Guiana* OR Guyan* OR Guyan* OR Paraguay* OR Paraguay* OR Peru* OR Surinam* OR Surinam* OR Uruguay* OR Venez* OR Belize* OR Costa Ric* OR Costarric* OR Costaric* OR Costa Ric* OR Costarric* OR Salvador* OR Salvador* OR El Salvador OR Guatemal* OR Guatemal* OR Guatemala OR Hondur* OR Nicaragu* Panam* OR Mexic* OR Cuba* OR Dominic* OR Dominic* OR Haiti* OR Jamaic* OR Puerto Ric* OR Puertorric* OR Puertoric*) |  |
|  | S48 | AB (Latin America* OR Latinamerica* OR Latinoamerica* OR Latin* OR Hispanic Americans OR Iberoamerica* OR Ibero Americ* OR Panamerican* OR Central America* OR Centroamerica* OR Mesoamerica* OR Meso America* OR Middle America* OR South America* OR Southamerica* OR Sudamerica* OR America del sur OR Caribbean OR Caribe* OR West Indi* OR Antill* OR Amerindian* OR Indians OR American Indian* OR Native America* OR Patagoni* OR Andes OR Andean* OR Amazon* OR Argentin* OR Bolivia* OR Brazil* OR Brasil* Colombia* OR Colombia* OR Colombia OR Chile* OR Ecuador* OR Guiana* OR Guyan* OR Guyan* OR Paraguay* OR Paraguay* OR Peru* OR Surinam* OR Surinam* OR Uruguay* OR Venez* OR Belize* OR Costa Ric* OR Costarric* OR Costaric* OR Costa Ric* OR Costarric* OR Salvador* OR Salvador* OR El Salvador OR Guatemal* OR Guatemal* OR Guatemala OR Hondur* OR Nicaragu* Panam* OR Mexic* OR Cuba* OR Dominic* OR Dominic* OR Haiti* OR Jamaic* OR Puerto Ric* OR Puertorric* OR Puertoric*) | 106,647 |
|  | S49 | S47 OR S48 | 109,862 |
|  | S50 | S21 AND S46 AND S49 Limiters - Published Date: 20100101-20200731 | 329 |

| **Database** | **Scopus**  **Date:** July, 17 2020 | | **Results** |
| --- | --- | --- | --- |
| **Search Strategy** | #1 | ( TITLE-ABS-KEY ( oral AND health ) OR TITLE-ABS-KEY ( mouth AND disease* ) OR TITLE-ABS-KEY ( tooth* ) OR TITLE-ABS-KEY ( dental ) OR TITLE-ABS-KEY ( teeth ) OR TITLE-ABS-KEY ( caries ) OR TITLE-ABS-KEY ( denture ) OR TITLE-ABS-KEY ( periodont* ) OR TITLE-ABS-KEY ( oral AND cancer ) OR TITLE-ABS-KEY ( mouth AND cancer ) OR TITLE-ABS-KEY ( oral AND tumor* ) OR TITLE-ABS-KEY ( mouth AND tumor* ) OR TITLE-ABS-KEY ( gingivit* ) OR TITLE-ABS-KEY ( malocclusion* ) OR TITLE-ABS-KEY ( gingival AND disease* ) OR TITLE-ABS-KEY ( crossbite* ) ) AND ( TITLE-ABS-KEY ( value AND of AND life ) OR TITLE-ABS-KEY ( quality AND of AND life ) OR TITLE-ABS-KEY ( quality-adjusted AND life AND years ) OR TITLE-ABS-KEY ( daly* ) OR TITLE-ABS-KEY ( qaly* ) OR TITLE-ABS-KEY ( quality-adjusted ) OR TITLE-ABS-KEY ( related AND quality ) OR TITLE-ABS-KEY ( hrqol ) OR TITLE-ABS-KEY ( qol ) OR TITLE-ABS-KEY ( dental AND impact ) OR TITLE-ABS-KEY ( social AND impact ) OR TITLE-ABS-KEY ( health AND impact ) OR TITLE-ABS-KEY ( cohqol ) OR TITLE-ABS-KEY ( child-oidp ) OR TITLE-ABS-KEY ( oqlq ) OR TITLE-ABS-KEY ( ohqol ) OR TITLE-ABS-KEY ( didl ) OR TITLE-ABS-KEY ( ecohis ) OR TITLE-ABS-KEY ( cpq* ) OR TITLE-ABS-KEY ( ohip ) OR TITLE-ABS-KEY ( gohai ) ) | 52,391 |
|  | #2 | TITLE-ABS-KEY ( "latin america" OR "Latinoamerica" OR latin* OR "central america" OR "Centroamerica" OR "south America" OR sudamerica OR caribbean OR caribe* OR "west indies" OR antill* OR patagoni* OR andes OR andean OR amazon OR "Puerto rico" OR puertoric* OR puertorric* OR jamaica OR jamaic* OR haiti OR haiti* OR "dominican republic" OR dominica* OR cuba OR cuba* OR mexico OR mexic* OR mejic* OR panama OR panam* OR nicaragua OR nicaragu* OR honduras OR hondur* OR guatemala OR guatemal* OR "el Salvador" OR salvador* OR "costa rica" OR costarric* OR costaric* OR belize OR beliz* OR venezuela OR venez* OR uruguay OR uruguay* OR suriname OR surinam* OR peru OR peru* OR paraguay OR paraguay* OR guyana OR guyan* OR "french guiana" OR guiana* OR guayan* OR ecuador OR ecuador* OR chile OR chile* OR colombia OR colombia* OR brazil OR brazil* OR brasil* OR bolivia OR bolivia* OR argentina OR argentin* | 1,314,513 |
|  | #3 | #1 AND #2 (2010 to July 17, 2020) | 1,679 |

| **Database** | **LILACS**  **Date:** July, 17 2020 | | **Results** |
| --- | --- | --- | --- |
| **Search Strategy** | #1 | (MH Oral Health OR MH Mouth Diseases OR Tooth$ OR Dental OR Teeth OR Odontológic$ OR Caries OR Denture$ OR Dentadura$ OR Periodont$ OR Oral OR MH Gingivitis OR Gingivit$ OR MH Malocclusion OR Malocclusion$ OR Gingival OR Maloclusión OR Má Oclusão OR Crossbite) AND (MH Value of Life OR MH Quality of Life OR MH Quality-Adjusted Life Years OR DALY$ OR QALY$ OR Quality-Adjusted OR Related-Quality OR HRQOL OR QoL OR Calidad-De-Vida OR Quality-Of-Life OR Dental-Impact OR Social-Impact OR Health-Impact OR COHQOL OR OIDP OR OQLQ OR OHQoL OR DIDL OR ECOHIS OR CPQ$ OR OHIP OR GOHAI) [Words] and 2010 OR 2011 OR 2012 OR 2013 OR 2014 OR 2015 OR 2016 OR 2017 OR 2018 OR 2019 OR 2020 [Country, year publication] | 834 |
